# Soil arthropods in bioindication and ecotoxicological approach: The case of the extreme environment Mefite (Ansanto Valley, Southern Italy)

**DOI:** 10.1016/j.heliyon.2024.e36342

**Published:** 2024-08-14

**Authors:** Sara Remelli, Tiziana Danise, Loris Galli, Cristina Menta

**Affiliations:** a Department of Chemistry, Life Sciences and Environmental Sustainability, University of Parma, Viale delle Scienze 11/A, 43124, Parma, Italy; b Department of Biotechnology, University of Verona, Strada le Grazie 15, 37134, Verona, Italy; c Department of Earth, Environment and Life Sciences, University of Genova, Corso Europa 26, 16132, Genova, Italy

**Keywords:** Soil acidity, *L. sativum*, *F. candida*, Collembola families, Protura species

## Abstract

Soil arthropods are pivotal in maintaining soil health and serve as sensitive indicators of soil alterations. The soil arthropod community in the Mefite Geological Site (Italy), characterized by a sulphurous lake and intense degassing, was the focus of this study. In details, the objectives were: i) to characterize soil arthropod community at different distances from the Mefite lake; ii) to identify resilient taxa acting as bioindicators to assess soil ecotoxicity. Soil cores were collected at A) 30m, B) 80m, C) 120m away from the lake; soil organic matter (SOM), and pH, ecotoxicity tests (*Lepidium sativum*: germination index, elongation inhibition; *Folsomia candida:* survival, reproduction), and identification of soil arthropods (orders, Collembola families, Protura species) have been carried out. Statistical analyses assessed the impact of sulphurous emissions on soil chemistry, ecotoxicity, and arthropod parameters (community structure, taxa associations, biodiversity indices like Shannon and Simpson, and soil biological quality index – QBS-ar). The results showed: no SOM differences; pH: A < B < C; the highest ecotoxic effects were observed in A for both target species; arthropod community composition and QBS-ar varied notably in A compared to C, with the lowest soil biodiversity found in A. Hypogastruridae (Collembola) showed a clear association with A, while Protura were notably absent in A. This study also provided the first records of 4 Protura species in Campania, updating existing knowledge. Overall, arthropod community biodiversity and composition proved to be effective soil bioindicators in highly acidic conditions, reflecting soil ecotoxicity. In particular, the QBS-ar index demonstrated sensitivity in sulphurous environments.

## Highlights

• Natural CO_2_ emission induces soil acidification and increases toxic effects.
• Arthropod communities reflect the CO_2_-concentration trend.
• Soil biodiversity decreases in extreme environments.
• Collembola show varied sensitivity and tolerance to disturbances.
• Proturan species list was updated for the Campania region.

## Graphical abstract

**Figure:**
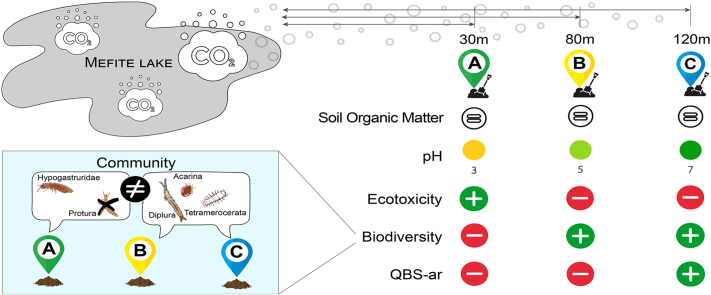

## 1 Introduction

Soil is one of the most complex and diverse ecosystems on earth, serving as the primary environmental foundation for a wide array of life forms, ranging from microorganisms to plants and animals [1,2]. Within the soil profile, the top 30 cm harbours a dense concentration of biological activity, coinciding with the highest levels of organic material [3,4]. This layer is a hub for soil invertebrate activities, which directly and indirectly influence various physical, chemical, and microbiological soil attributes, shaping ecosystem functioning and services [5,6]. Soil fauna, living in soil macropores and spaces in the soil-litter interface, is often studied for her representativeness in the evaluation of soil health. It is involved in many processes such as fragmentation and decomposition of organic material, water regulation, and participate in the biogeochemical cycles of carbon, nitrogen, phosphorus, and sulphur [[7], [8], [9]]. Moreover, soil fauna regulates microbial activity (including the control of pathogens) and distribution along soil profile, building tunnels, galleries, and other structures that facilitate organic matter incorporation and root growth [10,11]. Soil arthropods, a key component of soil fauna, besides playing a pivotal role in maintaining soil quality and health, also serve as effective indicators of soil quality.

Numerous studies utilize chemical indicators, such as pH and soil organic matter (SOM), as environmental impact indicators for terrestrial ecosystems, however, the impact of disturbances on soil may be more easily identified through their effects on soil living community [12,13]. Generally chemical and physical properties take longer than biological processes to be changed, and are less sensitive in detecting soil disturbances [14]. Consequently, assessing soil stressors via their effects on biota can offer early warnings of risks to terrestrial ecosystems [3]. As known, primary biomonitoring methods can be active, introducing organisms into the soil under controlled conditions and passive, observing and analysing indigenous ecosystem organisms [15]. Within active biomonitoring, invertebrates ecotoxicological tests are one of the first steps for soil risk assessments, and they can be applied to evaluate the biota safe exposure and to determine whether soil disturbances affect ecosystem services [13]. The outcomes of ecotoxicological tests depend on the environment characteristics and on the organism used in the bioassay [16,17]. Thus, the use of multiple target species, especially if representative of different trophic levels, is highly recommended, since considers different exposure routes and endpoints (e.g. survival, reproduction, growth and development) [[18], [19], [20]].

Within passive bioindicators, native soil fauna (with different endpoints, e.g. abundance, species richness, biological indices of either individual taxa, or of the entire community) have been used as indicator of different kind of impacts on terrestrial ecosystems because they are strictly correlated with physical, chemical, and microbiological soil attributes [5,21]. Strong adaptation to soil conditions makes some groups particularly sensitive to soil changes and thus indicators of soil quality; however, within soil arthropods, there are also some groups that can cope with soil disturbances. Indeed, soil animals inhabit a variety of extreme environments, where they must face habitat characterized by harsh and extreme conditions, beyond the optimal range for the development of life. The study of the native arthropod community living in extreme environments (e.g. saline meadows, deserts, Antarctic soils, and salt marshes, post-volcanic mofette fields) can help to understand the responses of soil arthropods to major soil disturbances and how those arthropods manage to survive the extreme conditions. Despite numerous laboratory experiments illustrating the short-term effects of CO_2_ on individual species under constant conditions (e.g., Ref. [22,23]), few field studies have investigated the responses of soil fauna to high CO₂ concentrations spanning from decades to millennia [24,25]. The Mefite Geological Site (Campania, Italy) host the largest (non-volcanic) natural emission of low temperature CO₂ rich gases ever measured, with a sulphurous lake which degassing affects the vegetation cover in the vast 3 km radius, causing toxic effects of CO₂, O₂ deficiency and high soil acidity [26]. With these unique characteristics, Mefite presents an opportunity to measure the effects of an extensive gas leakage.

This study aims to: (i) detect soil chemical parameters (SOM and pH) to investigate soil conditions in a degassing area, (ii) characterize native soil arthropod community at different distance from the Mefite lake; (iii) evaluate soil ecotoxicity, through active and passive bioindicators, employing a multi-organisms and multi-endpoints approach involving various taxonomic groups (*Lepidium sativum* for higher plants and *Folsomia candida* for mesofauna), (iv) identify soil arthropod taxa able to thrive in such an extreme environment.

## 2 Materials and methods

### 2.1 Study area

**Fig. 1:**
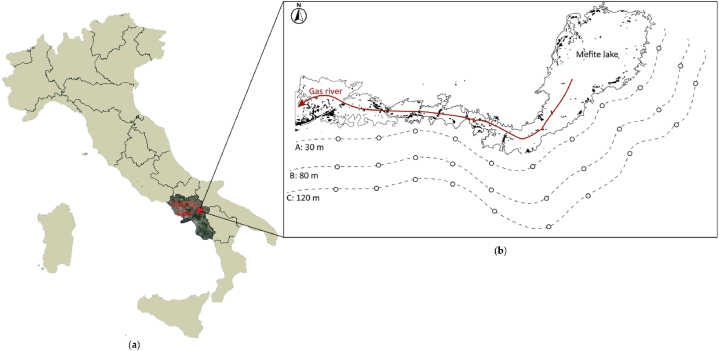
**(a)** Italy regions, with Campania (dark region) and the location of Mefite lake (red point); and **(b)** Mefite lake and the gas river (red arrow), with sampling distances (A: 30 m, B: 80 m, C: 120 m) from the lake indicated with dotted lines and sample points indicated with points.

Mefite is located in the Ansanto Valley (Campania, Southern Italy; Fig. 1a).

The site is included in a low hills system of Irpinia with pyroclastic overburdens characterised by soils with a sandy texture on the surface, moderately silty or silty in depth, developed on ash and fall ash pumice (Luvic-vitric Andosol) [27].

The area is characterized by the presence of vents and small boiling mud lakes that emit gases, among which the most abundant is CO_2_, that are probably linked to the presence of active fault systems responsible for the large historical earthquakes in the region [28,29]. The main emission area is at the foot of a landslide deposit, and consists of a bubbling mud pool, called “Mefite lake” or the “Gray Lake” (N 40°58′27.93″, E 15°8′45.06″), where the vegetation is absent or heavily damaged by the proximity of the degassing area. Mefite is the largest non-volcanic gas emission measured on the Earth with an estimated total CO_2_ flux of ∼2000 tons per day (98 % CO₂ + 1.3 % non-atmospheric N₂ + 0.33 % H₂S + 0.23 % CH₄)[28]. From the emission site, under low-wind conditions, the gas flows, due to its density, along a narrow natural channel producing a persistent invisible and lethal gas river [29]. Battaglini & Totaro Aloj [30] observed that the soil surrounding the bubbling mud has a low pH (<2.15 and 2.50), with a strong chalkysolfifera component and considerable concentration of sulphates, phosphates, nitrates, calcium, and ammonium. In the immediate vicinity of the lake the vegetation is profoundly affected by the extreme conditions and few plant species are able to survive, mainly *Phragmites australis* (Cav.) Trin. Ex Steud. As well as *Agrostis canina* L., and some of these present specific adaptations to the environment [31]. Along the gas river, it is important to identify an area where the landscape is gas-burnt with an absence of vegetation and where, at a distance of ca. 30 m, as the crow flies from the lake and river, there is mixed forest.

### 2.2 Sampling phase

The soil samples were collected between 24th and October 26, 2022 (Autumn), since the best period to take soil samples is away from dry period that can cause vertical migration, immobilization and aestivation of soil microarthropods [32]. Soil cores (10x10 × 10 cm) were sampled, starting from vegetation line, at 3 distances from both lake and river: A) 30 m, B) 80 m, C) 120 m as the crow flies (Fig. 1b); at each distance, 10 soil replicates ca. 40 m apart as the crow flies from each otherwhere collected (Fig. 1b). Cover vegetation was a mixed forest characterised by several plant groups including: oaks, dogwoods, blackthorns, poplars, maple and robinia, olms, ferns, grass, *Rosa canina*, straws, *Agrostis* sp., *Prunus* sp., brambles. The samples taken were kept at room temperature and carefully transported to the laboratory, avoiding compression and consequent damage to the pedofauna.

### 2.3 Chemical analyses

Soil samples were air dried and sieved, through a 2 mm mesh, before analyses. The determination of SOM was carried out using the Loss on Ignition (LOI) method, where 6 g of soil was subjected to drying at 105 °C for 24 h followed by ignition at 400 °C for 4 h [33]. For pH analysis, a soil:distilled water mixture at a ratio of 1:2.5 (w/v) was prepared, and the pH was measured using a pH meter equipped with automatic temperature compensation [34].

### 2.4 Ecotoxicological tests

Soil sample toxicity assessments were conducted utilizing *Folsomia candida* (Collembola: Isotomidae) and *Lepidium sativum* (Brassicales: Brassicaceae) as respectively representatives of soil invertebrates and plants. Before initiating the test procedure, soil samples (from which soil arthropods were previously extracted) were dried at 105 °C for 24 h, sieved at 2 mm, and homogenized.

For the ecotoxicological assessment of *F. candida*, Petri dishes were filled with 30 g of testing soil, moistened with deionized water to achieve 40–60 % of the total water holding capacity (WHC). *F. candida* specimens, utilized in the assays, were sourced from laboratory cultures at Parma University. Growth, survival and reproduction tests adhered to established ISO [35] protocols. Individual specimens were maintained at a temperature of 20 ± 2 °C and fed weekly with dry yeast. To obtain age-synchronized juveniles for testing purposes, specimens designated for egg deposition were collected from various breeding containers and mixed to ensure genetic diversity. All *F. candida,* used in the tests, were precisely 10 days old. Age synchronization was achieved by collecting eggs from deposition cultures and, immediately after hatching, introducing the juveniles into Petri dishes containing a moistened breeding substrate comprised of plaster of Paris and activated carbon powder (8:1 w/w). Ten age-synchronized *F. candida* individuals were introduced into each test Petri dish using an exhauster, ensuring no mortality occurred during the transfer. The springtails were maintained at 20 ± 2 °C and were provided with the same dry yeast utilized during breeding. Petri dishes were then incubated for 28 days, with weekly aeration and watering when water loss surpassed 2 % of the initial WHC. At the conclusion of the incubation period, the vessels were filled with water and gently agitated with a spatula to allow the springtails to float to the surface (floatation technique). To enhance visibility, approximately 0.5 mL of black ink was added to the water, facilitating the counting of surviving adults and new-born springtails using image analysis software, specifically ImageJ (version 1.53). The same methodology was applied to the control soil, utilizing standard [36] soil as a reference.

For the evaluation of soil toxicity, non-treated *L. sativum* seeds were employed to assess germination and root elongation, in accordance with OECD [37] procedures. Soil samples were tested by placing 15 g of testing soil in 9-cm diameter disposable Petri dishes, covered with Whatman #1 filter paper moistened with 5 mL of deionized water [38]. Ten undamaged seeds were introduced into each Petri dish, with all dishes hermetically sealed using ParafilmTM to prevent cross-contamination, and subsequently incubated in darkness at 25 ± 1 °C for 72 h. Following this incubation period, germinated seeds were tallied and root elongation (measured from the root tip to the radicle) was quantified. To ascertain the validity of the assay, the same methodology was applied using a control setup with Whatman #1 filter paper moistened with 5 mL of deionized water.

The elongation inhibition rate (EI%) was computed utilizing the following formula:

$$\text{EI}\%=\left(L_{c}\;–\;L_{n}\right)\;/\;L_{c}\;*\;100$$

where L_c_ and L_n_ were the mean values of root length in the control and in soil, respectively.

The germination index was calculated using the formula:

$$\text{GI}\%=\left(G_{n}\;*\;L_{n}\right)\;/\;\left(G_{c}\;*\;L_{c}\right)\;*\;100$$

where G_n_ and L_n_ were the mean values of germinated seeds and root in soil, respectively, and G_c_ and L_c_ were the mean values of germinated seeds and root in the control, respectively.

### 2.5 Arthropod community characterization

Soil microarthropods (with 200 μm^−2^ mm body width) were extracted from each soil sample using a Kempson extractor (ecoTech, Bonn, Germany). Extraction was run for 12 days, reaching 35 °C on day one, with 5 °C steps/24 h, until reaching 50 °C. The extracted specimens were collected and preserved in a solution of ethyl alcohol:glycerol (3:1). All arthropods were identified to the order level (subclass for Acarina, separating Oribatida as a function of their close relationship with soil organic matter); Collembola and Protura were further identified to the family and species level, respectively. Arthropods orders and Collembola families were identified and counted using an 8x–50x stereomicroscope (Zeiss Stemi SV6). Protura were cleared in lactic acid and mounted on slides in Marc André 2 medium, then they were identified to species and life stage levels with the aid of an interference contrast microscope (Leica DM LB2). Shannon index (H’) and Simpson index (D) were applied to the data obtained from the arthropods orders identification. To define soil biological quality was applied the QBS-ar index [39]. This index (i.e., biological soil quality based on arthropods) is based on soil microarthropod morphological features, assigning at each taxon, an Eco-Morphological index (EMI), ranging between 1 (=no adaptation) and 20 (=total adaptation) in relation to the adaptation level to soil. QBS-ar results from the sum of each maximum EMI score assigned at each taxon identified in the soil sample (details for the method are described in Ref. [32,39]). For the QBS-ar application, within holometabolous insects, adults and larvae were considered separately.

### 2.6 Statistical analysis

Non-parametric analyses were carried out on both chemical and arthropod parameters, since they do not meet the assumptions for parametric tests.

The Factor Analysis of Mixed Data (FAMD) was conducted using both quantitative and qualitative variables. The quantitative variables included pH, SOM content, total abundance of organisms, the number of taxa (N), biodiversity indices (Shannon and Simpson), and QBS-ar index. These variables were analyzed to determine their contribution to the overall variability observed in the study. Additionally, qualitative variables such as the distances from Mefite Lake (A, B, and C) were also included in the analysis to assess their influence on the variability observed in the dataset. FAMD data were computed and presented using the *FactoMineR* and *factoextra* packages, respectively; quantitative and qualitative variables are normalized during the analysis to balance the influence of each set of variables. Then, a correlation matrix using Spearman's Rank-Order Correlation was used to explore relationships between the multiple quantitative variables; the output was visualized using the *corrplot* package. The Kruskal–Wallis test was performed for multiple comparisons, followed by the Dunn's test pairwise comparisons between each independent Distance.

For arthropod orders and Collembola families assemblages analysis, the community matrix was square-root-transformed to reduce the relative influence of the most frequent orders, then non-metric multidimensional scaling (NMDS), based on Bray-Curtis dissimilarity index, followed by a permutational multivariate analysis of variance (PERMANOVA), was performed to visualize how patterns above illustrated influenced the grouping of arthropods communities. Taxa that were associated with a particular Distance, and the statistical significance of the association, were determined using a permutation test with the *multipatt* function from the *indicspecies* package. Using *multipatt*, the functions applied were: “IndVal.g”, to calculate Indicator Values for taxa, considering multiple groups and determining their specificity and sensitivity, and “r.g” function to calculate the relative abundance of a taxa in a group compared to its overall abundance in the dataset. Samples did not contain sufficient Protura individuals per species for applying inferential analysis, so that only descriptive analysis was applied.

A p-value ≤0.05 was considered significant. All analyses were performed using R (v.4.4.0 alpha) [40].

## 3 Results

### 3.1 Impact of distance on chemical parameters and arthropod indicators

**Fig. 2:**
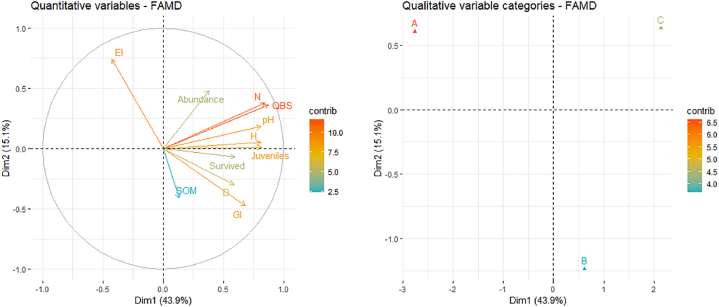
FAMD output for quantitative (chemical parameters: SOM-soil organic matter and pH; ecotoxicological parameters: *F. candida* Survived and Juveniles, and *L. sativum* GI-Germination Index and EI-Elongation inhibition; arthropods N-number of taxa and Abundance; biodiversity indices: H-Shannon and D-Simpson; and QBS-ar index) and qualitative (distances from Mefite lake: A-30 m, B-80 m, C-120 m) variables. The graphs of variables show the relationship between variables, the quality of the representation of variables, and the correlation between variables and the dimensions.

From the exploratory analysis conducted on the chemical/ecotoxicological parameters and arthropods indices investigated emerged an overall convergence of results (with the first dimension retaining most of the proportion of variance, Fig. 2).

**Fig. 3:**
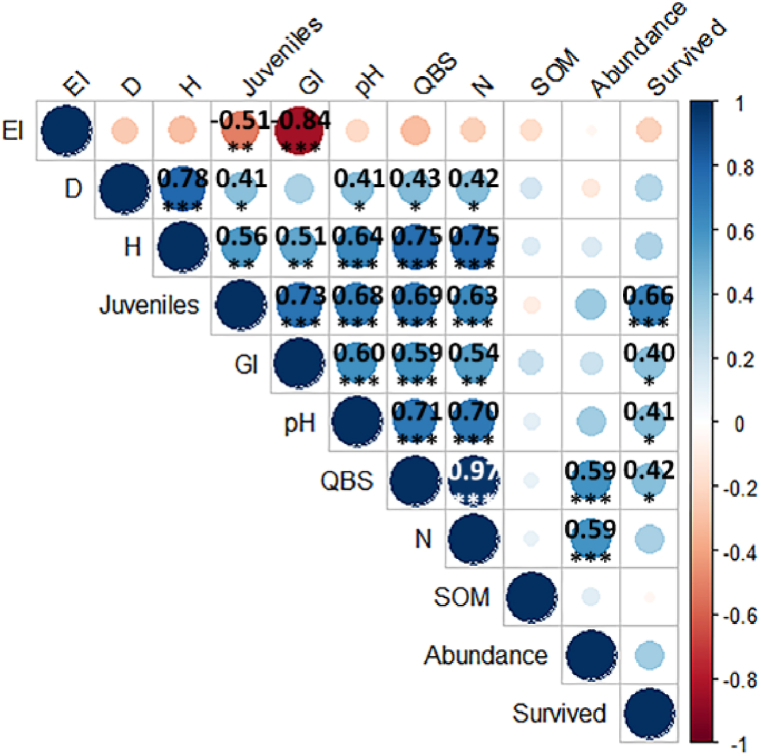
The correlogram visualizes the calculated correlation coefficients for the variables. Ecotoxicological parameters: *F. candida* Survived and Juveniles, and *L. sativum* GI-Germination Index and EI-Elongation inhibition; arthropods N-number of taxa and Abundance; biodiversity indices: H-Shannon and D-Simpson; and QBS-ar index). For significant correlations the exact Spearman coefficient is reported, together with the level of significance: ***p ≤ 0.001, **p ≤ 0.01, *p ≤ 0.05.

Variables that contribute the most to the first dimension are: pH, *F. candida* reproduction, number of arthropods groups and QBS-ar value, H’ index and abundance, and all show a trend to increase in C, while variables that contribute the most to the second dimension are: SOM content, *F. candida* survival, *L. sativum* GI and root elongation, and D index, all of them tending to increase in B. However, SOM content resulted the variable that contributed less to the principal dimensions, contrarily to QBS-ar value (and the number of groups to which is strictly correlated; see Fig. 3).

SOM did not correlate with the other variables, and EI was only negatively correlated with GI. Except for the just mentioned variables, pH and QBS-ar (and thus the number of groups) resulted significantly correlated to all the variables.

Results for each variable and their response, depending on the distance from Mefite lake, are reported below.

#### 3.1.1 Chemical analysis

The pH value varied from one distance to another (Fig. 2, p < 0.001), and was higher in C than in A and B (p < 0.001 and p < 0.05, respectively; Table 1), but it was also higher in B than in A (p < 0.05); soil organic matter (SOM) content did not differ significantly between the three distances even if it proves the lowest value in A (Table 1).

**Table 1:** Mean ± st.err. of chemical parameters at each distance (A: 30 m, B: 80 m, C: 120 m) from Mefite lake.

	A	B	C
pH	3.34 ± 0.15	5.18 ± 0.56	7.10 ± 0.32
SOM (%)	8.87 % ± 1.44 %	11.54 % ± 1.53 %	10.60 % ± 1.44 %

#### 3.1.2 Ecotoxicological tests

**Fig. 4:**
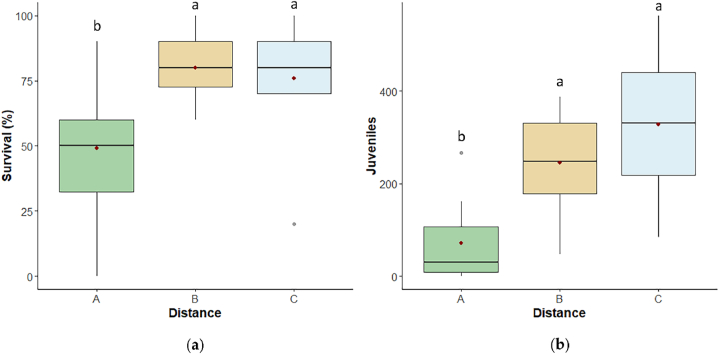
Boxplots of *F. candida***(a)** survival and **(b)** reproduction (n° of juveniles) after 28 days in A-30 m, B-80 m, C-120 m soils. The bottom and top of each box represent respectively the lower and upper quartiles; the line inside each box shows the median, the red cross shows the mean, and the whiskers indicate minimal and maximum observations. Different letters above the box of the same condition mean a significant difference (p ≤ 0.05) among the three distances.

**Fig. 5:**
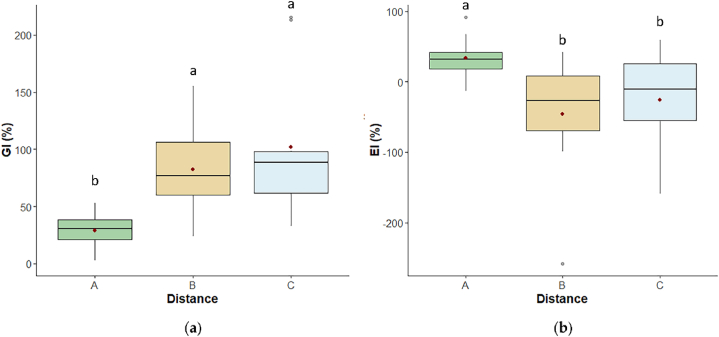
Boxplots of L. *sativum***(a)** germination index (GI) and **(b)** elongation inhibition (EI) after 72 h in A-30 m, B-80 m, C-120 m soils. The bottom and top of each box represent respectively the lower and upper quartiles; the line inside each box shows the median, the red cross shows the mean, and the whiskers indicate minimal and maximum observations. Different letters above the box of the same condition mean a significant difference (p ≤ 0.05) among the three distances.

Results of the ecotoxicological test on the *F. candida* and *L. sativum* target organisms gave the same trend (Fig. 4, Fig. 5).

*F. candida* survival and reproduction differed among distances (p < 0.05 and p ≤ 0.001, respectively), and resulted higher in B and C than in A (survival: p < 0.05, both; reproduction: p ≤ 0.01 and p < 0.001, respectively) (Fig. 4a and b), with survival data in A showing the highest variability. The reproduction was the endpoint with the highest sensitivity to soil conditions at the different distances from Mefite lake.

*L. sativum* data supported *F. candida* results showing the highest soil ecotoxicity in A. Indeed, GI varied significantly among distances (p < 0.001), being lower in A than in B and C (p ≤ 0.001, both), and the same applied to EI (p < 0.05), which reported the highest values in A (that means that root elongation was higher in B and C than in A; p < 0.001 and p ≤ 0.05, respectively)(Fig. 5a–b).

For both endpoints a higher variability was observed in B and C than in A, with a higher sensitivity to the distance from Mefite lake for germination results. Root elongation often resulted higher in B and C than in the control test (see the negative values of EI%).

#### 3.1.3 Arthropods community

Total arthropods abundance, despite not being significantly different compared to the three distances together, showed differences in pairwise comparisons, being higher in C than in A and B (p ≤ 0.05, both; Table 2).

**Table 2:** Mean ± st.err. (ind./m^2^) and relative abundance (%) of arthropods orders (Acarina subclass) at each distance (A-30 m, B-80 m, C-120 m) from Mefite lake. Different letters mean significant differences (p ≤ 0.05), no letters mean a statistical similarity.

		A			%	B			%	C			%
Arachnida	Acarina	150.18	±	48.48b	8.19	493.85	±	201.86b	25.71	828.86	±	119.00a	31.26
	- Oribatida	25.99	±	11.75b		161.7	±	69.04 ab		332.1	±	94.35°	
	Araneae	2.89	±	2.89	0.16	2.89	±	2.89	0.15	23.10	±	10.37	0.87
	Palpigrada		–				–			2.89	±	2.89	0.11
	Pseudoscorpionida	8.66	±	6.16	0.47		–				–		
Chilopoda	Geophilomorpha	2.89	±	2.89	0.16	5.78	±	3.85	0.30	8.66	±	6.16	0.33
	Lithobiomorpha		–			8.66	±	4.41	0.45	8.66	±	6.16	0.33
	Scolopendromorpha		–			2.89	±	2.89	0.15		–		
Diplopoda	Glomerida		–				–			2.89	±	2.89	0.11
	Julida		–				–			2.89	±	2.89	0.11
	Polyxenida		–			20.22	±	12.21	1.05	20.22	±	12.21	0.76
Pauropoda	Tetramerocerata	5.78	±	3.85b	0.31	31.77	±	20.40b	1.65	103.97	±	37.83°	3.92
Symphyla	Symphyla	51.98	±	26.82b	2.83	184.83	±	81.25 ab	9.62	132.85	±	22.86°	5.01
Malacostraca	Isopoda		–			8.66	±	8.66	0.45	5.78	±	3.85	0.22
Entognatha	Collembola	1389.13	±	603.05	75.75	730.66	±	323.15	38.05	1094.55	±	200.52	41.29
	Diplura	11.55	±	11.55b	0.63	46.21	±	26.61 ab	2.41	63.54	±	19.63°	2.40
	Protura		–	b		54.87	±	36.90°	2.86	31.77	±	17.46°	1.20
Insecta	Coleoptera	72.20	±	22.88b	3.94	190.61	±	47.98 ab	9.92	228.15	±	38.85°	8.61
	- larvae	69.31	±	21.18		158.84	±	37.59		202.16	±	40.61	
	Diptera	14.44	±	7.76b	0.79	25.99	±	10.05 ab	1.35	40.43	±	7.70°	1.53
	- larvae	5.78	±	3.85		17.33	±	9.82		31.77	±	9.08	
	Hemiptera	25.99	±	25.99	1.42	5.78	±	3.85	0.30	8.66	±	6.16	0.33
	- Cicada larvae		–			2.89	±	2.89			–		
	Hymenoptera	98.19	±	52.41	5.35	106.86	±	65.86	5.56	37.54	±	12.95	1.42
	- larvae	8.66	±	4.41		11.55	±	8.82		5.78	±	3.85	
	Lepidoptera larvae		–				–			2.89	±	2.89	0.11
	Thysanoptera		–				–			2.89	±	2.89	0.11
	N° of taxa	4.4	±	0.64b		6.60	±	1.01b		9.50	±	0.72°	
	Abundance (ind./m^2^)	2044.70	±	635.05b		2249.75	±	721.74b		2965.98	±	265.39°	

Marked differences were observed for the number of taxa (p ≤ 0.001), which was the highest in C (p < 0.001 compared to A, and p ≤ 0.05 compared to B; Table 2).

**Fig. 6:**
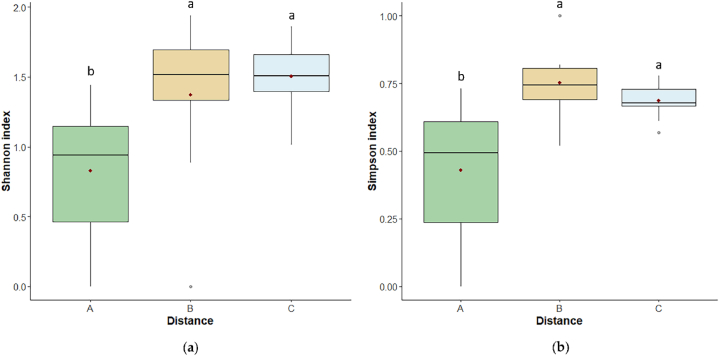
Boxplots of biodiversity indices **(a)** Shannon index and **(b)** Simpson index at A-30 m, B-80 m, C-120 m distances. The bottom and top of each box represent respectively the lower and upper quartiles; the line inside each box shows the median, the red cross shows the mean, and the whiskers indicate minimal and maximum observations. Different letters above the box of the same condition mean a significant difference (p ≤ 0.05) among the three distances.

Biodiversity indices responded similarly to the distance from the Mefite lake, indeed both differed significantly among distances (Shannon index: p < 0.01, Simpson index: p ≤ 0.001; Fig. 6a–b).

For both indices, A hosted the lowest biodiversity (Shannon: p < 0.01 compared to both B and C; Simpson: p < 0.001 compared to B, and p ≤ 0.01 compared to C).

**Fig. 7:**
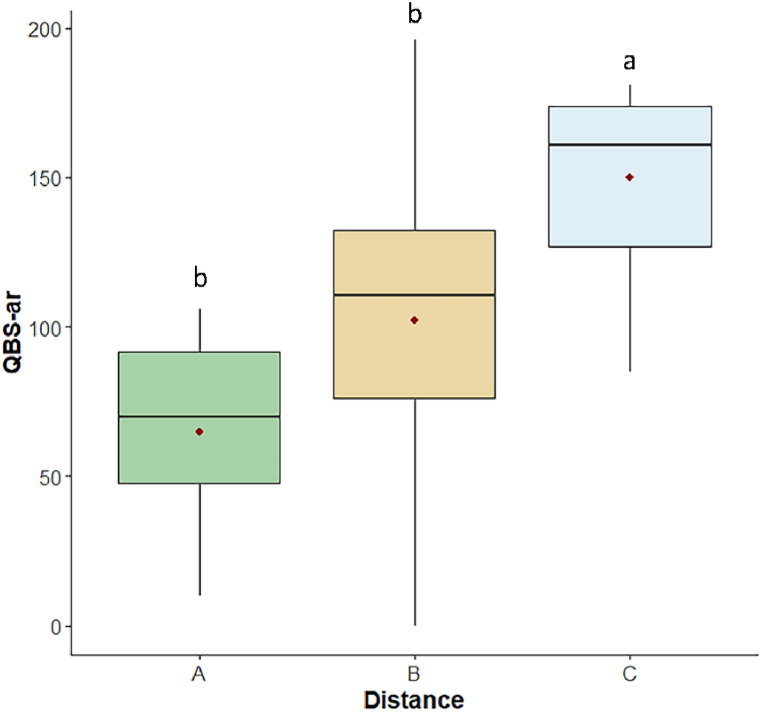
Boxplots of QBS-ar index at A-30 m, B-80 m, C-120 m distances. The bottom and top of each box represent respectively the lower and upper quartiles; the line inside each box shows the median, the red cross shows the mean, and the whiskers indicate minimal and maximum observations. Different letters above the box of the same condition mean a significant difference (p ≤ 0.05) among the three distances.

Soil biological quality index based on soil arthropods QBS-ar differed among distance (p < 0.001; Fig. 7), with C showing higher results than A and B (p < 0.001 and p < 0.05 respectively).

### 3.2 Impact of distance on arthropods community structure

Examining the results obtained on soil arthropod community, Collembola showed the major relative abundance (50 %), followed by Acarina (23 %). The other larger groups were: Coleoptera (8 %), Symphyla (6 %), Hymenoptera (4 %), Tetramerocerata (2 %), Diplura (2 %), Protura and Diptera (1 %, both); each of the remaining taxa (Table 2) represented less than 1 % of arthropod community. Those proportion reflected the communities in B and C, even if in C Tetramerocerata, Diplura and Diptera were more abundant than Hymenoptera. In A, instead, Hymenoptera were more abundant than Coleoptera and Symphyla; while Diptera, Diplura and Tetramerocerata accounted each one for less than 1 % of the population (0.79 %, 0.63 % and 0.31 %, respectively), with Protura being absent. Acarina/Collembola ratio differed between distances (p < 0.01) and, despite being always less than 1, resulted significantly lower in A than in B and C (p < 0.01, both).

#### 3.2.1 Arthropods community

**Fig. 8:**
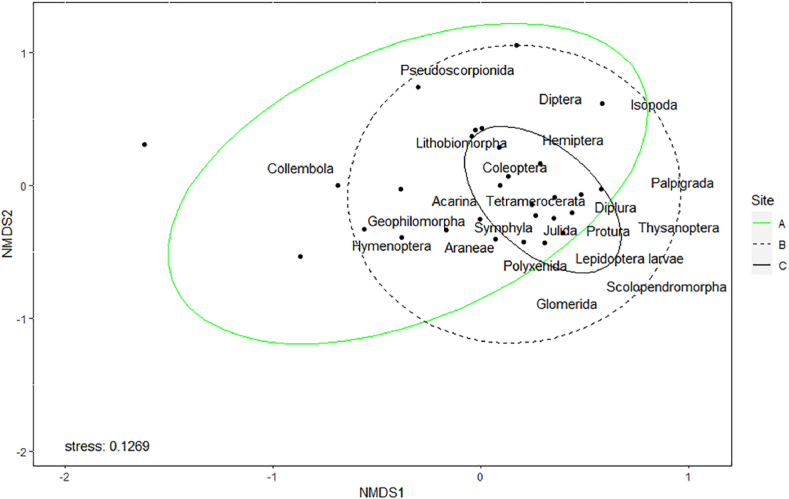
Bray–Curtis-based NMDS plot of arthropod community composition at the three distances (A-30 m, B-80 m, C-120 m). Points represent samples.

Investigating arthropods assemblages at the three distances, community structure resulted related to distance from Mefite lake (p ≤ 0.01), specifically with the highest difference between A and C (p < 0.01, Fig. 8), regardless of SOM content and pH.

Tetramerocerata resulted an indicator taxon associated to C distance (IndVal.g: p < 0.01), with a specificity of 71.68 % (i.e the probability that the surveyed site belongs to the target site group given the fact that the taxa has been found) and a sensitivity of 90.00 % (i.e the probability of finding the taxa in sites belonging to the site group), and showed also a strong correlation with this site (r.g. function: p < 0.05). Acarina (IndVal.g: p < 0.001), Symphyla and Diplura (IndVal.g: p < 0.05) associated with both B and C, with a specificity of 90.17 %, 86.68 % and 90.86 % respectively, and a sensitivity of 100 %, 78.95 % and 63.16 % respectively. However, only Acarina and Coleoptera resulted in a significantly higher relative abundance in B and C than in A (r.g: p ≤ 0.01, both). Only four out of 22 taxa tend not to avoid A: Pseudoscorpionida, Collembola, Hemiptera, and Hymenoptera. The relative abundance of a taxon in a group, as well as the results of pairwise comparisons among distances for each taxon absolute abundance, are shown in Table 2.

#### 3.2.2 Collembola community

In the study area, the most abundant Collembola family was Isotomidae (41 %), followed by Hypogastruridae (19 %), Onichiuridae and Ciphoderidae (10 %, both) (Table 3).

**Table 3:** Mean ± st.err. (ind./m^2^) and relative abundance (%) of Collembola families at each distance (A-30 m, B-80 m, C-120 m) from Mefite lake. Different letters mean significant differences (p ≤ 0.05), no letters mean a statistical similarity.

	A			%	B			%	C			%
Arropalitidae		–			5.78	±	3.85	0.79	5.78	±	3.85	0.53
Bourletiellidae		–				–			5.78	±	5.78	0.53
Ciphoderidae	31.77	±	17.46	2.29	98.19	±	61.37	13.44	184.83	±	105.56	16.89
Entomobryidae	49.10	±	36.03	3.53	112.63	±	68.54	15.42	103.97	±	48.36	9.50
Hypogastruridae	482.30	±	311.61°	34.72	69.31	±	54.15b	9.49	60.65	±	42.06 ab	5.54
Isotomidae	635.36	±	304.88	45.74	320.57	±	239.49	43.87	349.45	±	108.44	31.93
Neanuridae		–			5.78	±	5.78	0.79	8.66	±	6.16	0.79
Neelidae	34.66	±	34.66	2.49	0.00	–	0.00		5.78	±	3.85	0.53
Odontellidae	75.09	±	44.99	5.41	2.89	±	2.89	0.40	46.21	±	19.35	4.22
Onychiuridae	23.10	±	20.10	1.66	86.64	±	58.40	11.86	207.94	±	140.55	19.00
Sminturidae	2.89	±	2.89	0.21	5.78	±	3.85	0.79		–		
Tullbergiidae	54.87	±	32.35 ab	3.95	23.10	±	20.10b	3.16	115.52	±	50.57°	10.55

Entomobryidae, Tullbergiidae, Odontellidae and Neelidae accounted for 8 %, 6 %, 4 % and 1 % of the Collembola, respectively. Each of the remaining families represented less than 1 % of the assemblage.

Collembola family structure did not change significantly among sites at different distance, nor depending on pH or SOM content, and no families were indicators (IndVal.g: p > 0.05) of specific distances. Hypogastruridae resulted correlated with A (r.g: p < 0.05), and only four out of 12 families tended not to avoid A: Hypogastruridae, Isotomidae, Neelidae, and Odontellidae. The relative abundance of Collembola families at each distance, as well as the results of pairwise comparisons among distances for each family absolute abundance, are shown in Table 3.

#### 3.2.3 Protura community

A total of 28 proturans were identified at species level, for a total of 5 species (Table 4): 53 % *Acerentomon microrhinus*, 29 % *Acerentulus traegardhi*, 11 % *Eosentomon armatum*, and 4 % *Proturentomon* sp.

**Table 4:** Proturan species (row number) at each distance (B and C, no Protura were found in A-0m from Mefite lake).

	B				C			
	Female	Male	Maturus Junior	%	Female	Male	Maturus Junior	%
*Acerentomon microrhinus* Berlese, 1909	8	5		68	1	1		22
*Acerentulus confinis*	1			5				
*Acerentulus traegardhi* Ionesco, 1937	1		1	11	2	3	1	67
*Eosentomon armatum* Stach, 1926	2			11	1			11
*Proturentomon* sp.^a^	1			5				

a species was not identified due to the poor preservation.

Three species, *Acerentulus traegardhi*, *Acerentomon microrhinus* and *Eosentomon armatum*, and genus *Proturentomon*, were reported for the first time in Campania based on this study [41].

In B, that is at intermediate distance from the Mefite lake, a higher species richness (5 in B, 3 in C) and an unbalanced sex ratio (2.6:1 in B, 1:1 in C) were observed.

## 4 Discussion

### 4.1 Chemical parameters and arthropods indicators

The study aimed to assess the impact of an extreme environment characterized by CO_2_-rich degassing vents and highly acidic soils on edaphic arthropods. Active and passive bioindicators were employed through laboratory and field studies, respectively, to evaluate the influence of these conditions, aligning with chemical data. Additionally, ecotoxicological tests included representatives of plant components to provide further insights into soil toxicity. The investigation area, recognized as the largest non-volcanic natural emission of low-temperature CO_2_-rich gases, represents a unique field laboratory in which the effect of long-term high concentrations of CO_2_ on natural ecosystems can be assessed [42,43]. These sites make it possible to assess the long-term effects of high CO_2_ concentrations on natural ecosystems, potentially reflecting future CO_2_ levels [31]. The well-established effects of CO_2_ on soil, including its impact on vegetation absence or damage, are compounded by the concurrent presence of H_2_S, leading to the formation of substantial amounts of H_2_SO_4_ and subsequent low pH in the surrounding soil [28,44,45]. This extreme environment possesses the potential to exert edaphic selection on soil communities, as highlighted by previous observations by Di Iorio et al. [31]. Our study supported the previous observation, indeed pH resulted highly correlated with almost all the variable observed, with the only exception being SOM, elongation inhibition, and total arthropods abundance. The correlation between soil pH and SOM is generally context-dependent and can vary. On one hand, a lot of authors [[46], [47], [48]] reported that low pH had a negative impact on SOM, but on the other, high CO_2_ levels come with restricted decomposition (due to the exclusion of much of both the vegetation and soil biota) and can thus enhance SOM concentrations compared to reference soils [49]. In this study, no relation was observed between SOM and pH, and SOM did not vary among 30m, 80m and 120m from Mefite lake, suggesting that the effects on SOM of soil extreme acidity and CO_2_ levels could counterbalance each other.

Even if pH was probably the most determining factor based on ecotoxicological tests, due to the evaporation of the gases in the soil during the transport to laboratory, no correlation was observed with elongation inhibition of *L. sativum*, which yet resulted enhanced in A soils. This could be explained by the *L. sativum* sensitivity to highly acidic soil (indeed, under conditions of strong soil acidiﬁcation, all plants stopped growing at pH values below 3; [50,51]), confirmed also by germination index results. On the contrary, mildly acid and neutral soils, are all suitable for *L. sativum* growth. Elongation parameter resulted correlated not only with germination but also with its soil fauna counterpart, *F. candida* reproduction, emphasizing the interconnectedness of ecological responses. *F. candida* reproduction is generally a more sensitive parameter than survival [20], but these soils showed similar outcomes in ecotoxicological effects both for test organisms and endpoints. It is worth noting that not only the ecotoxicological results of the target species belonging to different trophic levels coincide, but also the results of arthropod indices, the latter being influenced not only by pH acidity but also by the exposure in the field to the degassing vents. In this study, it was observed that both biodiversity indices, Shannon and Simpson, exhibited a negative response in proximity to Mefite Lake. This observation suggests that soil arthropod communities experienced a reduction in overall biodiversity and an increase in the dominance of select arthropod orders. Notably, the sensitivity of these indices did not differentiate between the intermediate distance (B) and C. In contrast, the QBS-ar index appeared more susceptible to environmental disturbances such as pH acidity and CO_2_ concentrations, potentially indicating a heightened sensitivity to these factors compared to Shannon and Simpson indices. In this study, QBS-ar index showed a strong correlation with *F. candida* (both endpoints) and *L. sativum* germination, highlighting the potential of the index to reflect soil stressors in extreme environments. Nevertheless, results are in accordance with Fernández-Montiel et al. [52], who observed that increasing CO_2_ flux affected negatively the abundance and richness of soil biota, for both micro-organisms and mesofauna.

### 4.2 Arthropods communities

Even if a few studies were conducted near Mefite lake, mostly on plants [31,53], studies on soil fauna are lacking also in other CO_2_-rich environments (e.g. mofettes), with Collembola being the most investigated taxon [24,25].

As explained above, the major environmental drivers in the Mefite system are CO_2_ and soil acidity, and those parameters are expected to influence soil fauna favouring adapted species and excluding acid-sensitive species [24]. Dominant species belonged to the Collembola and Acarina groups, with the Acarina/Collembola ratio always less than 1, suggesting low-quality soils [54] and confirming a soil degradation condition near Mefite lake. Regarding soil arthropods community structure, soil acidification played a less prominent role in explaining species’ distributions than expected; a similar result was obtained by observing Collembola and Nematoda communities in mofette soils [24]. Tetramerocerata were about 6 times more abundant in B than in A, and 3 times more abundant in C than in B, confirming their high sensitivity to environmental stress. Although with less marked differences between A and B, in this study Symphyla and Diplura resulted also indicators of habitat disturbance. However, as observed by Zsolt Tóth et al. [55], Tetramerocerata, Symphyla and Diplura (unlike Protura) are able to survive in more disturbed soils (like A ones), despite the fact that these groups generally avoid soil disturbance [9]. On the other hand, Acarina can show different specific responses to soil alteration. Generally, the dominance of Oribatida increases with acidification, but this was not observed in the present study; however, species abundances are often pH interval-dependant and, in the case of this study, changes in competition and food availability could have been a key factor [56]. Coleoptera high relative abundances in B and C confirm the results of Ghiglieno et al. [57], which observed an association of this taxon with higher pH values.

Despite the evidence of site A being a hostile environment for soil arthropods, some of them seemed not to be affected by these extreme conditions. Pseudoscorpionida, strangely collected only in A, are considered relevant predators and regulators of the density of small soil arthropods [58,59], and we can hypothesize that the distribution of this group depends more on prey availability than environmental variables. In addition, most of Hymenoptera (here mostly Formicidae) and Hemiptera species are very mobile fauna, and this could be a possible explanation to their lack of sensitivity to the present soil disturbances.

In this study, Collembola resulted not only ubiquitous but also slightly more abundant in A, possibly because of the significant variability of responses to food resources, soil temperature, moisture, and chemical properties of the different species [11,57,60]. Species living in extreme environmental conditions are expected to cope better, not only because they are physiologically adapted to the physicochemical extremes of the habitat, but also because they benefit from CO_2_ [23,25]. As observed by Russell et al. [25], due to its toxic nature, direct benefits from CO_2_ extreme concentrations, like those observed at Mefite, could be excluded; however, this group can benefit from CO_2_-induced alterations in nutrient resources and/or from competition-free niches. The same authors suggested that Collembola, dwelling in air-filled soil pores, have a less direct contact with acidification and are probably more affected via their food resources. This observation resulted in contrast to what observed in this study, at least for the target species *F. candida*, yet acidity was the main factor driving soil toxicity at the laboratory scale. Hypogastruridae correlation with site A supported the hypothesis that this family can withstand a variety of soil disturbances, indeed it was shown to be the dominant family also in contaminated sites (e.g. by PAH and by PCB and PCDD/F) [20,61]. As observed by Kuznetzova [62], under conditions of environmental stress it often occurs that the greater abundance of few tolerant species results into large total densities of springtails. This could be the case of Isotomidae, which resistance to high CO_2_ concentrations, like those found in mofettes, is well-known at least for one species, *Folsomia mofettophila* sp. nov. [63,63]).

Proturan high sensitivity to disturbances is well known, indeed it is a group characterised by high adaptation to soil life, typical of stable environments and undisturbed soils, and it was observed to be highly sensitive to soil geochemistry and environmental changes that impact its food resources (mainly mycorrhizae) [64,65]. Galli, Capurro, Molyneux et al. [66] evidenced a negative correlation between pH values and proturan densities, result that seems to be in contrast with this study. A possible explanation of this apparent incongruity can be that in Galli's study [66] pH ranged between 5.9 and 7.3, while in this study the pH lowest value was much more acidic. Indeed, a higher abundance, species richness and unbalanced sex ratio (common in Protura populations; [66]) was found at intermediate distance from the Mefite lake, where pH mean value was around 5.18, suggesting a possible preference of this group for slightly acidic soils, and thus a preferential range of pH as already evidenced by Galli, Capurro, Molyneux et al. [66]. Regarding the unbalanced sex ratio, Galli, Capurro, Molyneux et al. [66] observed that, even if it appears to be a common feature among Protura populations, it was detected predominantly in Acerontomata species but not in *Eosentomon* genus, in contrast to what observed in the present study. Nevertheless, Protura presence in Campania is scarcely recorded (first records belong to Ref. [67,68]), reporting *Acerentulus confinis* as the dominant species, here only present in one specimen at 80m from the lake.

At the end, it is worth noting that based on this study three species of Protura (*Acerentulus traegardhi, Acerentomon microrhinus* and *Eosentomon armatum*) and genus *Proturentomon* were recorded for the first time in Campania [41].

## 5 Conclusion

This study extends the actual knowledge on the impact on soil arthropods of long-term exposure to extreme CO_2_ concentrations, both directly and indirectly (e.g. through soil acidification). The ecotoxicological results highlighted the scale of the impact produced, primarily by pH, on target organisms not only belonging to soil arthropod fauna, but also to plants, suggesting the potential cascade effect that the degassing vents can generate on the ecosystem. In addition to ecotoxicological data information, arthropod communities extracted from the field confirm CO_2_ and soil acidity as the major environmental drivers of the Mefite system.

At order level, arthropod community structure resulted a sensitive soil bioindicator in CO₂ rich environments, reflecting soil biological quality community. Arthropod community responded to extreme conditions with a decrease in biodiversity as well as in the abundance of well-adapted taxa, like Tetramerocerata who confirmed to prefer more structured soils. Nevertheless, although no orders/subclass resulted significantly associated with the most disturbed soils, some taxa showed to be able to cope with such environmental extremes, being possibly adapted to these conditions, or via an altered food supply and/or lack of competitors. We can suppose that in some cases, the lack of significance in the association could be due to the high variability of responses within the order level. Indeed, deepening into the Collembola order, it was observed that even if some families, like Onychiuridae, were sensitive to the proximity of the Mefite lake, other families tolerate or even increased their abundance in the most disturbed area. Protura's high sensitivity to environmental disturbance is well known; however, higher species richness at intermediate distance from the Mefite lake suggested that their responses to soil conditions could be strongly linked to a preferential range of pH. At the same time species-specific sex ratio responses to disturbances should be deepened. Finally, Protura presence in Campania is scarcely recorded, and this study updated the previous knowledge on this poorly known taxon, thus confirming the importance already underlined by Galli (2023) of handing over to taxonomists the specimens collected in collections for different purposes.

## Data availability statement

Data will be made available on request.

## CRediT authorship contribution statement

**Sara Remelli:** Writing – review & editing, Writing – original draft, Visualization, Validation, Investigation, Formal analysis, Data curation, Conceptualization. **Tiziana Danise:** Writing – review & editing, Investigation, Conceptualization. **Loris Galli:** Writing – review & editing, Investigation. **Cristina Menta:** Writing – review & editing, Writing – original draft, Validation, Supervision, Project administration, Funding acquisition, Conceptualization.

## Declaration of competing interest

The authors declare that they have no known competing financial interests or personal relationships that could have appeared to influence the work reported in this paper.
